# The Utility of a Small Animal Grip Strength Measurement Device as a Model for Studying Exercise-Induced Muscle Damage

**DOI:** 10.3390/antiox15010023

**Published:** 2025-12-23

**Authors:** Haruki Kobori, Jiapeng Huang, Yishan Tong, Shuo Wang, Cong Wu, Ziwei Zhang, Sihui Ma, Yasuhiro Seki, Llion Arwyn Roberts, Katsuhiko Suzuki

**Affiliations:** 1 Graduate School of Sport Sciences, Waseda University, Tokorozawa 359-1192, Japan; koboharu1223@fuji.waseda.jp (H.K.); hjpshidsg1234@toki.waseda.jp (J.H.); tongyishan130@ruri.waseda.jp (Y.T.); wang_sh@akane.waseda.jp (S.W.); wucong86@ruri.waseda.jp (C.W.); y-seki@wismerll.co.jp (Y.S.); 2 Faculty of Sport Sciences, Waseda University, Tokorozawa 359-1192, Japan; 3 Australian Centre for Precision Health and Technology, Griffith University, Gold Coast 4215, Australia; llion.roberts@griffith.edu.au

**Keywords:** exercise-induced muscle damage (EIMD), small animal grip strength measurement device, aldolase, oxidative stress, eccentric exercise, triceps brachii

## Abstract

Exercise-induced muscle damage (EIMD) is characterized by structural muscle tissue damage and elevated biochemical markers following high-intensity or unaccustomed exercise. This study evaluated the utility of a small animal grip strength measurement device as a model for EIMD. Thirty-four male mice were divided into four groups: one control and three experimental groups, and sacrificed at 2, 4, and 7 days post-exercise. The exercise protocol involved 50 tail-pull contractions at 60 Hz using a forelimb grip strength device. Biochemical biomarkers, inflammatory gene expression, and oxidative stress markers from blood and muscle tissue were assessed at each sacrificial time point. Muscle damage marker, plasma aldolase activity, showed significant elevation at 4 days post-exercise (*p* < 0.01). Inflammatory gene expression in triceps brachii showed no significant changes. Oxidative stress analysis revealed significantly decreased biological antioxidant potential (BAP) at 7 days and a trend toward a significant increase in Diacron-reactive oxygen metabolites (d-ROMs) at 4 days. NF-kB expression showed a trend toward significance increase. The grip strength exercise model induced modest biochemical alterations suggesting possible involvement of oxidative stress. The early release of aldolase and subsequent oxidative stress suggest that this model replicates EIMD and may serve as a valuable tool for quantitative loading on muscles, studying EIMD mechanisms and facilitating EIMD-based interventions.

## 1. Introduction

Exercise-induced muscle damage (EIMD) occurs due to high-intensity or unaccustomed exercise, typically associated with eccentric contractions. Generally, muscle damage is characterized by ultrastructural changes to muscle, like streaming, disruption, and dissolution of the Z-disk [1]. EIMD is also characterized by several key temporal changes, including increased creatine kinase (CK) and other muscle damage markers in blood levels, infiltration of inflammatory cells, and necrosis of muscle fibers [2].

High-intensity training or unaccustomed exercise also causes delayed-onset muscle soreness (DOMS). Generally, DOMS is the pain experienced some time after exercise. Underpinning DOMS, it is believed that breaking down muscle fibers and tearing the muscle membrane causes increased inflammation and pain, but the exact mechanisms of DOMS are unknown. Mizumura et al. referred to the B2 bradykinin receptor–nerve growth factor (NGF) pathway and COX-2-glial cell line pathway involved in DOMS. These two neurotrophic factors are produced by muscle fibers and/or satellite cells [3]. The degree of DOMS is often used as a muscle damage marker to measure the extent of muscle damage [4], but there is also a report that the extent of EIMD does not reflect the level of DOMS [5], and the finer details of their association remain unclear.

Eccentric contractions refer to the action of lengthening muscle fibers during muscle contraction, e.g., when lowering a dumbbell in training, the primary muscles involved act eccentrically. On the other hand, concentric contraction refers to the action of shortening fibers during muscle contraction. In concentric contraction, the muscle exerts motor actions, whereas in eccentric contraction, the muscle exerts motor-braking actions against the external load [6]. It is considered that eccentric contractions induce greater muscle damage than concentric contractions [7]. Proske and Allen reported that muscle force remains reduced for up to a week after eccentric contractions, but recovery was within 1–2 h after concentric contractions [8]. Muscle damage has also been confirmed with eccentric contractions histologically, allowing the viewing of ultrastructural changes [9].

When damage occurs to the body and. specifically, skeletal muscle in this instance, pro- and anti-inflammatory substances named cytokines are released [10]. These are released by muscle tissues and typically increase after exercise. The expression of pro-inflammatory cytokines like tumor necrosis factor-α (TNF-α) and interleukin (IL)-1β was observed in tissues [11]. In endurance exercise, inflammatory cytokines like TNF-α, IL-1β, and IL-6 were elevated after a marathon [12]; however, pro-inflammatory cytokine responses are variable. The inflammatory cytokines TNF-α and IL-6 can be elevated by a single bout of exertion but reduced by long-term regular exercise [13]. Exercise-induced muscle damage is closely associated with oxidative stress, and it is characterized by mechanical stress and subsequent oxidative stress [14,15]. The oxygen regularly inhaled is essential for ongoing ATP production within cells, a process primarily carried out by mitochondria. However, under these circumstances, not all oxygen is fully metabolized into carbon dioxide and water via the mitochondrial Krebs cycle and electron transport chain; a fraction of the oxygen is instead converted into reactive oxygen species (ROS). ROS, due to their unpaired electrons, exhibit high reactivity and tend to extract electrons from other normal molecules. This process, repeated in succession, initiates a chain reaction of oxidative events, and the resulting oxidative stress can damage DNA, inactivate enzymes, and promote inflammation [16,17]. Under normal conditions, ROS are maintained at concentrations that pose no harm to the organism, as the endogenous antioxidant systems effectively protect cells. However, during exercise, the increased oxygen demand can lead to elevated ROS production. If ROS levels exceed a certain threshold and surpass the capacity of the antioxidant defense systems, oxidative stress is caused.

Current animal models of EIMD and DOMS rely on, e.g., treadmill running [18,19], electrical stimulation [20,21,22], and toxin injection [23], each aligned with significant limitations. For example, treadmill protocols require exercise volumes substantially higher than completed by voluntary exercise, while introducing confounding stress responses. Electrical stimulation demonstrates extreme parameter variability (1–100 Hz) and artificial activation patterns that bypass normal motor recruitment. Finally, toxin injection creates muscle fiber destruction, compared to contraction-based fiber damage.

Resultingly, the artificial nature of damage induction often produces supra-physiological injury patterns that may not reflect normal exercise-induced adaptations, limiting translational relevance.

Functional assessment offers several advantages over traditional damage induction methods. Unlike an artificial injury model, functional approaches evaluate integrated muscle performance, providing outcome measures directly relevant to the physiological consequences of exercise-induced changes. Grip strength measurement specifically offers non-invasive assessment, longitudinal monitoring capability, and objective quantification that can be standardized across laboratories, as the eccentric contraction component controlled by contraction number could cause EIMD while maintaining physiological relevance. During tail-pulling resistance within rodent models, forearm muscles experience repetitive eccentric contractions similar to damage-inducing human exercise, but in a measurable format.

This study evaluated the utility of a small animal grip strength measurement device alongside tail pulling exercise as a novel EIMD model. We hypothesized that repetitive eccentric contractions during grip strength testing would produce measurable muscle damage that could be characterized by biochemical parameters, inflammatory gene expression, and oxidative stress responses. We examined whether this approach could address some of the methodological limitations of current models while maintaining physiological relevance.

## 2. Materials and Methods

### 2.1. Animals

Thirty-four male mice aged 8 to 9 weeks (C57BL/6NcrSLc) were purchased from Japan SLC (Shizuoka, Hamamatsu, Japan). All animals were allowed to adapt to the environment for a week. Mice were housed two per cage in a controlled environment under a 12 h light–dark cycle, and they were allowed free access to regular food and water throughout the experiments. The experimental procedure was approved by the Guiding Principles of Animal Care and Use of the Academic Research Ethics Review Committee of Waseda University (Approval No. A24-127, approved on 29 March 2024).

### 2.2. Hand Grip

Forelimb grip strength was measured using a specialized grip strength meter (Melquest, Toyama, Japan) originally designed for assessing muscle strength, growth, and neuromuscular function in rodents. The exercise protocol involved positioning mice on a horizontal platform and allowing them to grasp a metal bar with their forelimbs. Subsequently, the tail was pulled horizontally until grip failure occurred, at a controlled frequency of 60 Hz based on metronome pacing. Grip failure was defined as the moment when the mouse released its grip from the bar. Tails were pulled 50 times per animal to induce repetitive eccentric contractions of the forelimb muscles. The tail-pulling was performed manually by experienced investigators to maintain a consistent rhythm and force application. Peak grip force at the moment of each release was recorded using Toriemon USB software ver.1.02 (Nidec Drive Technology, Kyoto, Japan). Control animals were not handled in order to avoid inducing eccentric contraction and affecting biochemical markers.

**Small animal grip force measurement device.**

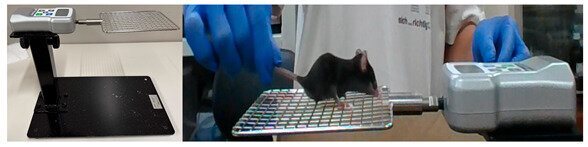

### 2.3. Experimental Grouping and Sample Collection

Mice were randomly divided into four sacrificial dissection groups: 2 days (*n* = 9), 4 days (*n* = 9), 7 days after exercise (*n* = 8), and the control group (*n* = 8). Mice were dissected after being sacrificed via the inhalant isoflurane (Abbott, Tokyo, Japan). The muscle tissues and organs were excised and frozen in liquid nitrogen and the duration between all tissue excision for each mouse and nitrogen freezing was approximately 15 min. The same investigator completed each type of tissue dissection. Blood samples of approximately 500 µL were collected from the abdominal artery using heparin as an anticoagulant. Plasma was obtained from blood samples by centrifugation at 1500× *g* for 15 min at 4 °C, producing about 300 µL of plasma per sample. Tissue samples were snap-frozen in liquid nitrogen, and blood and tissue samples were stored in a deep freezer at −80 °C until analysis.

### 2.4. Measurement of Plasma Biochemical Parameters

The plasma levels of aspartate aminotransferase (AST), alanine aminotransferase (ALT), creatine kinase (CK), uric acid (UA), blood urea nitrogen (BUN), creatinine (CRE), lactate dehydrogenase (LDH), and aldolase (ALD) were measured by Kotobiken Medical Laboratories (IBARAKI, Tsukuba, Japan).

### 2.5. Gene Expression Analysis

The total RNA of muscle was extracted using Nucleo Spin RNA (TAKARA, Kusatsu, Japan). The concentration of total RNA was assessed using the NanoDrop system (NanoDrop Technologies, Wilmington, DE, USA). Then, total RNA was reverse transcribed to cDNA using the Clontech PCR Thermal Cycler GP (TAKARA, Kusatsu, Japan) according to the manufacturer instructions.

Real-time qPCR was performed with the Thermal Cycler Dice Real Time System IV (TAKARA, Kusatsu, Japan) using the TB Green^®^ Premix Ex Taq™ II FAST qPCR (TAKARA, Kusatsu, Japan. Item No. RR830B (A × 2)). The thermal profiles consisted of 30 s at 95 °C for denaturation followed by 95 °C for 5 s and annealing at 60 °C for 10 s. A total of 18s mRNA was used as the housekeeping gene, and the ΔΔCT method was used to quantify target gene expression. All data were interpreted as fold change relative to the expression values of the control group at the matching time points. The primer sequences for real-time PCR of each target gene are detailed in Table 1.

### 2.6. Oxidative Stress Markers

Plasma biological antioxidant potential (BAP) test kit (Wismerll, Tokyo, Japan) was used per manufacturer instructions. Briefly, the premise is based on FeCl reacting with colorless thiocyanate to form Fe_3_^+^ ions, producing a red color [24]. The sample is then added and reduced to Fe_2_^+^ ions of antioxidants, and the red color is decolorized. The color change is measured and proportional to antioxidant content. Absorbance was measured at 505 nm, and results were expressed in μM/L. In advance, the machine was warmed to 37 °C and all cuvettes in the thermostat space for 10 min to warm up. After that, the cuvettes were quickly zero adjusted in the photometer. All cuvettes were subsequently filled with 50 µL of coloring solution, placed in the photometer, and the control was measured. Then, 10 µL of plasma sample was placed in the cuvettes, warmed in the thermostat space for 5 min, and the antioxidant capacity was measured in the photometer.

The plasma diacron-reactive oxygen metabolites (d-ROMs) test (Wismerll, Tokyo, Japan) measures oxidative stress in blood samples by mainly measuring hydroperoxide (ROOH).

In an acidic buffer, iron ions catalyze the conversion of hydroperoxides (ROOH) into radicals. Subsequently, these radicals oxidize N, N-diethyl-para-phenylenediamine (DPPD) to form a colored radical cation, which is measured photometrically [25]. Result was expressed in Carratelli units (U-CARR), and 1 U-CARR was defined as having the same oxidizing capacity as 0.08 mg H_2_O_2_/dL. In advance, the machine and all cuvettes in the thermostat space were warmed to 37 °C for 10 min. A total of 20 µL of plasma was placed in warmed reagent and mixed well. Then, 20 µL of the DPPD solution was added to the reagent with the plasma and mixed well. After mixing, the cuvette was placed in the photometer space and measured.

The plasma OXY-adsorbent test (Wismerll, Tokyo, Japan) measures total antioxidative capacity in blood based on the actions of hypochlorous acid (HOCl), one of the ROS produced by white blood that leads to oxidative stress [26]. By mixing the sample with the HOCl reagent, the antioxidant reaction against HOCl occurs and N, N-diethyl-para-phenylenediamine (DPPD) is added. The HOCl that could not be removed by the sample reacts with DPPD, allowing the antioxidant capacity to be assessed. Absorbance was measured at 505 nm, and results were expressed in μM/mL. At first, cuvettes containing distilled water were placed in the photometer for zero adjustment. The reagent with HOCl was put into empty cuvettes including control cuvettes. Then, the machine was set to 37 °C and all cuvettes were put in the thermostat space to keep them warm for 10 min. A total of 10 µL of plasma and a tube containing 1 mL distilled water were mixed to prepare diluted samples. After temperature control, 10 µL of the diluted samples was added to the cuvettes and mixed. Then, the mixed samples were placed in the thermostat space. In measuring, 10 µL of the DPPD solution was put into the cuvette which was prepared for the control, mixed, and placed in the photometer. After the control measurement was completed, 10 µL of the DPPD solution was put into the cuvette with samples, mixed, and measured in the photometer.

### 2.7. Data Analysis

All data were assessed by one-way analysis of variance (ANOVA) using the SPSS statistical analysis package IBM, SPSS Statistics 29.0.2 (Tokyo, Japan). When this analysis revealed a significant interaction, Tukey’s post hoc test was performed to determine the significance among the means. Furthermore, this study analyzed the associations between variables using Spearman correlation coefficient. The relationship between the r value and the effect strength is proposed as 0.1 < r < 0.3: small effect, weak correlation; 0.3 < r < 0.5: medium effect, moderate correlation; r > 0.5: large effect, strong correlation. Statistical significance was defined as *p* < 0.05, and a significant trend defined as *p* < 0.1. Most data error bars are presented as standard deviation (SD); the error bar for CK is shown as standard error (SE) due to the large variability in this measurement.

## 3. Results

### 3.1. Grip Strength Performance

**Figure 1:**
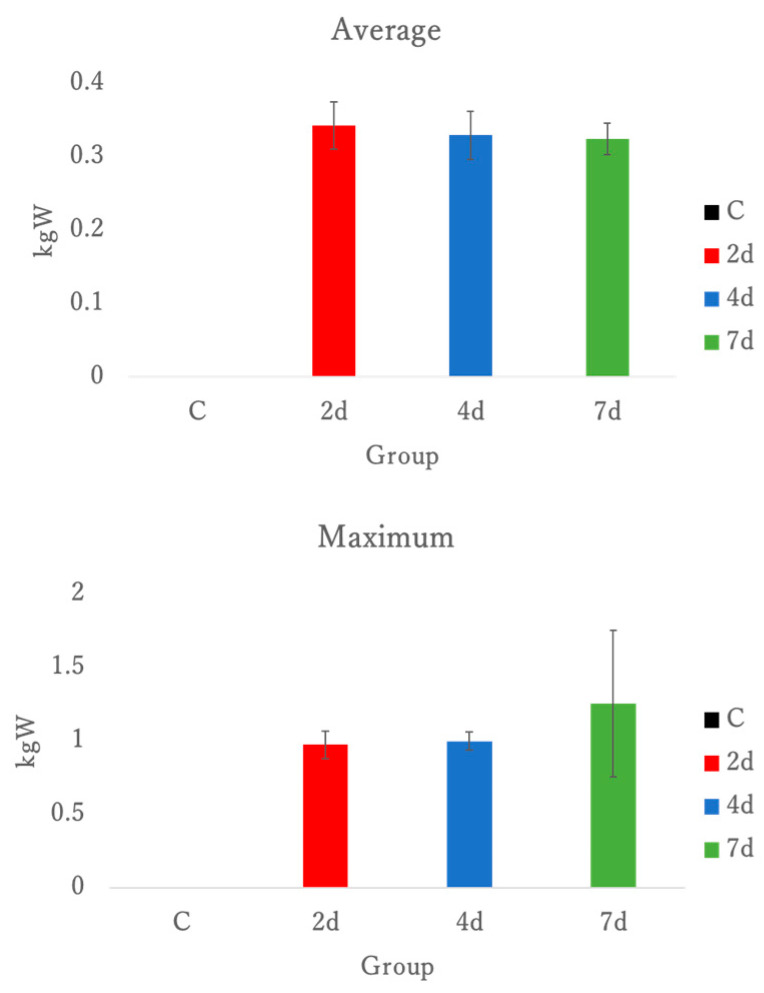
Average and maximum hand grip strength data; C, control; 2 d, 4 d, and 7 d, post-exercise dissection time.

All mouse tails were pulled 50 times per animal to induce eccentric contractions at a pace of 60 Hz based on metronome pacing. Notably, the control group was not subjected to any exercise load. Therefore, the data presented here focused on three groups dissected at 2, 4, and 7 days post-exercise. There was no significant difference in average or peak grip strength between the three groups. See Figure 1.

### 3.2. Muscle Damage Markers

**Figure 2:**
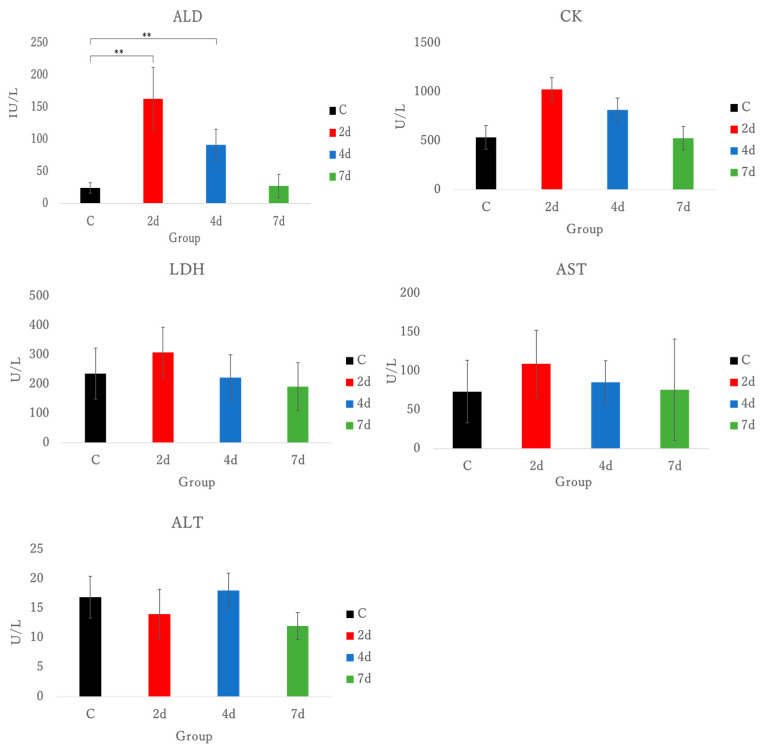
The activities of ALD, CK, LDH, AST, and ALT in plasma. ALD, aldolase; CK, creatin kinase; LDH, lactate dehydrogenase; AST, aspartate aminotransferase; ALT, alanine aminotransferase; C, control; 2 d, 4 d and 7 d, post-exercise dissection time; ** *p* < 0.01.

ALD was significantly elevated in the group that was dissected 2 and 4 days after exercise (*p* < 0.01). The activities of CK, LDH, and AST were elevated in the group that was dissected 2 days after exercise, but no significant differences were observed. See Figure 2.

**Figure 3:**
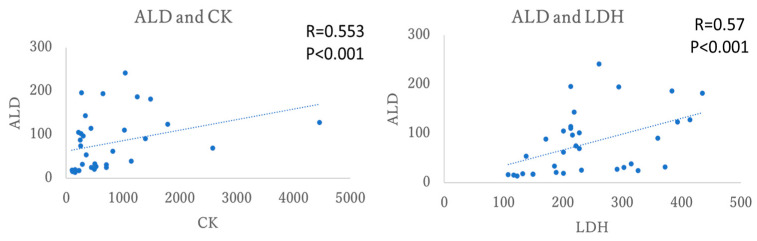
The correlation analysis with ALD, CK and LDH in mice plasma; ALD, aldolase; CK, creatin kinase; LDH, lactate dehydrogenase.

The increase in ALD observed in the groups at 2 d and 4 d post-exercise, correlated with CK and LDH. The results observed a significant moderate correlation between ALD and both CK and LDH. See Figure 3.

**Figure 4:**
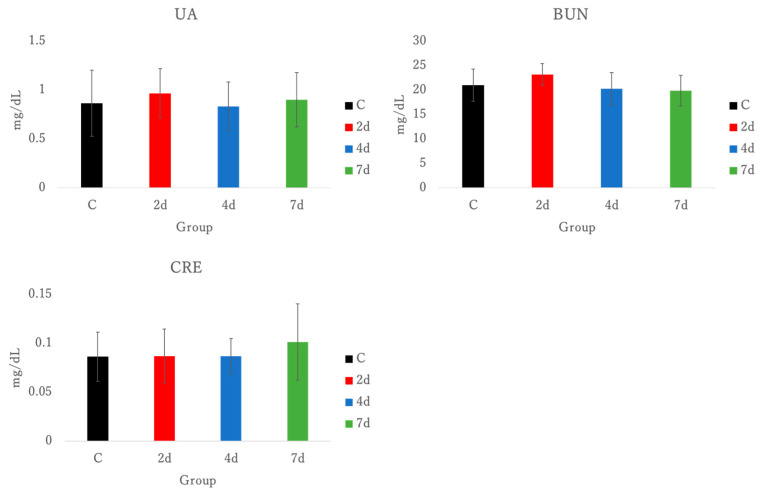
The concentration of UA, BUN, and CRE in plasma. UA, uric acid; BUN, blood urea nitrogen; CRE, creatinine; C, control; 2 d, 4 d, and 7 d, post-exercise dissection time.

Uric acid (UA), blood urea nitrogen (BUN), and creatinine (CRE) in plasma were measured to confirm whether kidney dysfunction has occurred, but no significant differences were observed. See Figure 4.

### 3.3. Muscle Damage-Related Gene Expression in Triceps Brachii

#### 3.3.1. Inflammation-Related Gene Expression in Triceps Brachii

**Figure 5:**
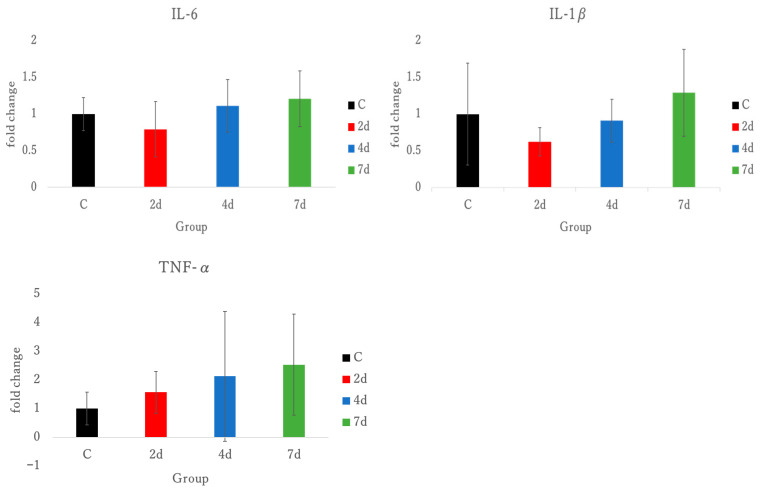
Inflammation-related gene expression due to muscle damage in triceps brachii, IL-6, interleukin 6; IL-1β, interleukin 1β; TNF-α, tumor necrosis factor-α; C, control; 2 d, 4 d, and 7 d, post-exercise dissection time.

Interleukin 6 (IL-6), IL-1β, tumor necrosis factor-α (TNF-α), and Ly6g were measured as pro-inflammatory markers in triceps brachii. Exercise with forelimb grip did not have significant effects on IL-6, IL-1β, and TNF-α. Also, exercise with forelimb grip could not be confirmed in Ly6g expression due to being below the threshold of the PCR machine. See Figure 5. 

#### 3.3.2. Anti-Inflammation-Related Cytokine Gene Expression Due to Muscle Damage in Triceps Brachii

IL-10 was measured as an anti-inflammatory cytokine in triceps brachii. Exercise with forelimb grip did not induce IL-10 expression.

#### 3.3.3. Oxidative Stress-Related Cytokine Gene Expression Due to Muscle Damage in Triceps Brachii

Nuclear factor kappa beta (NF-kB) and kelch-like ECH-associated protein 1 (Keap1) were measured as inflammatory biomarkers in triceps brachii. The statistical analysis did not reveal significant differences, but trends toward significance were observed (*p* = 0.088).

Keap1 showed no significant difference in gene expression.

**Figure 6:**
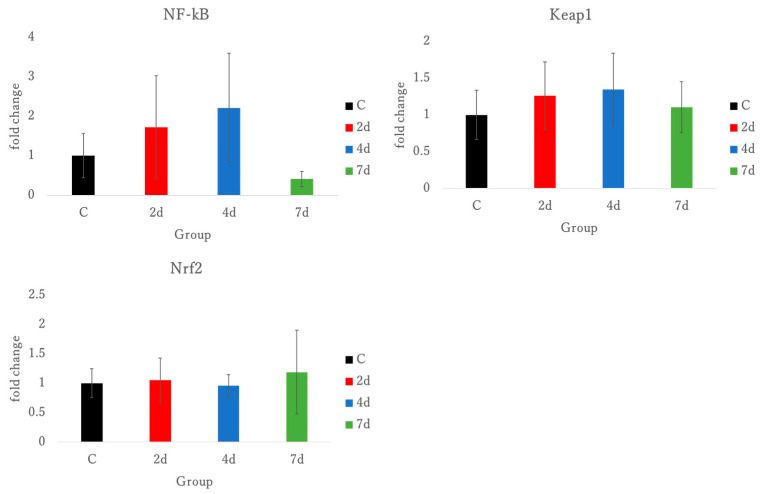
Oxidative stress-related gene expression due to muscle damage in triceps brachii. NF-kB, nuclear factor kappa B; Keap1, kelch-like ECH-associated protein 1; Nrf2, nuclear factor (erythroid-derived 2)-like 2; C, control; 2 d, 4 d, and 7 d, post-exercise dissection time.

Nuclear factor (erythroid-derived 2)-like 2 (Nrf2) was measured as an anti-inflammatory biomarker in triceps brachii. Exercise with forelimb grip did not induce a significant gene expression. See Figure 6.

### 3.4. Oxidative Stress Markers in Plasma

As a total antioxidant indicator, BAP decreased in the group dissected 7 days after exercise compared to the control group (*p* < 0.01) and the group dissected 4 days after exercise (*p* < 0.05). In the group dissected 7 days after exercise, no significant difference was observed as compared to 2 days, but a significant trend was observed (*p* = 0.062).

For d-ROMs, as an oxidative stress marker, the group dissected 4 days after exercise had no significant difference observed, but a significant trend was observed (*p* = 0.059).

**Figure 7:**
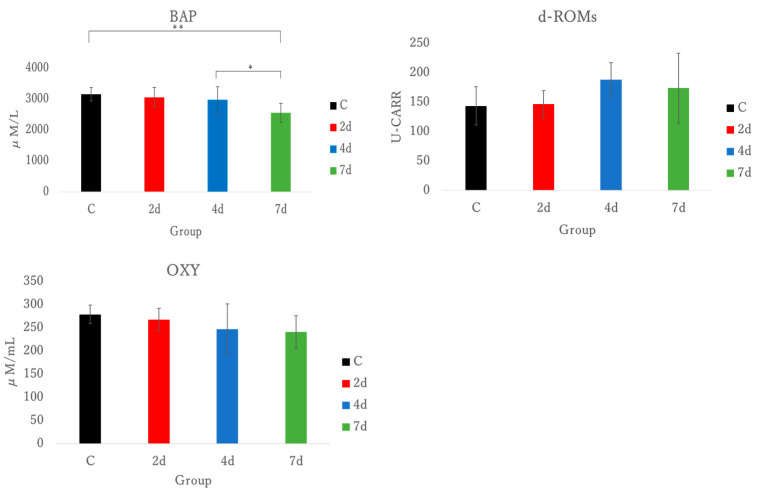
BAP test as total antioxidant indicator; BAP, biological antioxidant potential; d-ROMs as oxidative stress; d-ROMs, derivatives of reactive oxygen metabolites; OXY test as total antioxidative capacity; OXY, oxy-adsorbent test; C, control; 2 d, 4 d, and 7 d, post-exercise dissection time; * *p* < 0.05, ** *p* < 0.01.

OXY test was conducted as total antioxidative capacity, but no significant difference was observed. See Figure 7.

## 4. Discussion

In this study, we examined the usefulness of forelimb grip exercise as a tool to induce EIMD in mice, based on examining temporal post-exercise markers of inflammation, cell integrity, and oxidative stress. Plasma-AST, ALT, CK, UA, BUN, CRE, LDH, and ALD were measured, but no significant difference in AST, ALT, CK, UA, BUN, CRE, and LDH were observed (Figure 2, Figure 4). Whilst CK, LDH, and ALD are also used as markers of muscle damage [27], plasma ALD values were elevated in mice dissected 2 and 4 days after exercise here, when compared to the control group.

This study demonstrates that a grip strength measurement device can serve as a novel model for inducing EIMD, that produced measurable biochemical changes through oxidative stress pathways rather than classical inflammatory responses. The selective elevation of aldolase without concurrent increases in traditional markers, combined with delayed oxidative stress patterns, suggests a unique mechanistic profile that addresses several limitations of current EIMD models.

In EIMD scenarios, CK levels begin to increase 24 to 48 h following eccentric exercise and typically peak around 96 h post-exercise [28,29,30], postulated to appear within days and not the early hours, due to its molecular weight. Similarly, in animal models, CK levels also show an elevation following exercise [31]. The elevation of CK levels in mice typically peaks at 24 or 36 h post-exercise; however, no significant differences were observed in this study. Nevertheless, compared to the control group, the group dissected 2 days after exercise showed an increase in CK level. This suggests that the lack of significant differences might be attributed to the small sample size. The molecular weight of CK is about 80 kDa~82 kDa [32], whereas ALD is about 40 kDa [33], and the difference in molecular weight between CK and ALD suggests that the smaller molecular weight of ALD may contribute to its higher membrane permeability compared to CK and LDH, potentially leading to its earlier release into the bloodstream. Smaller cytoplasmic enzymes may be released earlier from damaged cells compared to larger proteins, suggesting our model produces controlled membrane disruption without extensive structural damage [27]. Aldolase has been used as a marker of muscle damage, but it is not as widely recognized or commonly utilized as CK or LDH. The moderate correlations between aldolase and both CK and LDH validate aldolase as a legitimate muscle damage marker while demonstrating mechanistic independence from traditional indicators (Figure 3).

In this study, TNF-α, IL-1β, and IL-6 were measured as inflammatory markers from the triceps brachii in exercise-induced muscle damage (Figure 5). IL-10 was measured as an anti-inflammatory marker. No significant differences were observed in the pro-inflammatory cytokines such as TNF-α, IL-1β, and IL-6. Typically, the levels of TNF-α, IL-1β, and IL-6 increase following intense exercise. TNF-α, IL-1β, and IL-6 are major inflammatory markers that contribute to the promotion of inflammatory responses after exercise [34]. Notably, TNF-α and IL-1β are characterized by their role in the degradation of damaged muscle tissue [35]. Previous studies have reported no significant differences in inflammatory mediators following eccentric contraction exercises [36,37]. In the exercise-loading model used in this study, no significant differences in inflammatory mediators were observed, consistent with the findings of these prior studies [36,37], thus contributing to the understanding of inflammatory mediators being minimal in eccentric EIMD.

In contrast, endurance exercises are associated with a marked increase in inflammatory cytokines. Brenner reported that prolonged exercise using a cycle ergometer under four experimental conditions significantly increased TNF-α and IL-6 in healthy male subjects [38]. Similarly, Suzuki et al. reported that the elevation of IL-6 persisted at 3 and 12 h post-exercise [39]. While endurance exercises prominently elevate post-exercise inflammatory mediators, resistance exercises do not appear to produce significant changes in these mediators. This suggests that the mechanisms of muscle damage between these two exercise modalities may differ. In this study, no significant differences in TNF-α, IL-1β, and IL-6 were observed using RT-PCR analysis. These findings suggest that inflammatory markers may not be useful indicators for characterizing muscle damage in this experiment.

To assess oxidative stress levels, this study used tests for BAP, d-ROMs, and OXY in plasma samples (Figure 7). While no significant differences were observed in d-ROMs and OXY levels, d-ROMs exhibited a significant trend. A significant decrease in BAP levels was observed in the 7 days group. Given that d-ROMs observed a trend toward significance at 4 days, oxidative stress reached its highest level, and as a result of the antioxidant response, antioxidant substances were depleted in the 7 days group, leading to a decrease in BAP. Consequently, RT-PCR was performed to measure NF-kB, Nrf2, and KEAP1, which are involved in inflammation and oxidative stress (Figure 6). Although no significant differences were identified for NF-kB, Nrf2, and KEAP1, a significant trend was observed for NF-kB. These findings suggest that the oxidative stress measurement by d-ROMs and the transcription factor NF-kB were associated with oxidative stress and inflammation, and exhibited significant trends. This indicates that the exercise-induced muscle damage model, using handgrip exercises, may induce oxidative stress. Oxidative stress can be a marker of muscle damage and can trigger tissue inflammation if it progresses. NF-kB and Nrf2 are transcription factors involved in the cellular oxidative stress response, regulating oxidative and inflammatory reactions as well as antioxidant responses in a reciprocal manner [11]. NF-kB responds to exercise-induced stress and promotes the expression of inflammatory cytokines in skeletal muscle [40]. However, in the exercise-induced muscle damage model using the handgrip in this study, no correlation was observed between NF-kB and TNF-α or IL-1β. Our model’s most distinctive feature is the production of oxidative stress response without concurrent inflammatory activation. The trend toward increased d-ROMs at 4 days followed by significant BAP depletion at 7 days suggests progressive antioxidant system exhaustion rather than acute oxidative burst.

KEAP1 is a protein that regulates the cellular oxidative stress response and acts as a suppressor of the transcription factor Nrf2 [41]. Under conditions of oxidative stress, the binding between KEAP1 and Nrf2 is disrupted, allowing Nrf2 to initiate the expression of antioxidant genes. In this study, RT-PCR analysis revealed no significant differences in the gene expression of Nrf2 and KEAP1. Furthermore, the antioxidant stress tests, including BAP and the OXY adsorption test, did not demonstrate antioxidant responses to oxidative stress.

The absence of inflammatory gene expression changes distinguishes this model from traditional EIMD approaches, which consistently demonstrate TNF-α, IL-1β, and IL-6 elevation within 2–6 h [42]. This dissociation may reflect damage below the threshold for inflammatory cell recruitment, or qualitatively different loading patterns compared to sustained eccentric exercise. The trend toward NF-kB activation without cytokine expression suggests oxidative stress signaling below the inflammatory threshold.

Therefore, the exercise-induced muscle damage model using a handgrip exercise applied in this study appears capable of inducing oxidative stress. This suggests that the model may trigger exercise-induced muscle damage via oxidative stress. The small animal grip strength measurement device used in this study was found to induce oxidative stress by horizontally pulling the mouse’s tail. However, because the experimenter pulls the tail manually, it was difficult to pull the same load to all mice and maintain a consistent tension. We would like to make it a research challenge going forward to determine how to pull a consistent load to mice. Although no antioxidant responses were observed in this study, it is possible that modifying the exercise intensity could elicit an antioxidant response to oxidative stress.

Compared to other established models, our approach offers several advantages. Unlike treadmill running requiring many times higher exercise volumes, or electrical stimulation with extreme parameter variability (1~100 Hz), grip strength testing provides standardized, quantifiable loading. Traditional models produce fiber destruction with recovery requiring many days, while our model shows controlled, reversible changes maintaining normal renal function.

The reproducibility potential addresses a major limitation in EIMD research, where multi-laboratory studies reveal significant outcome variability despite identical protocols. However, limitations include the manual tail-pulling procedure introducing force variability, and the absence of inflammatory activation limiting the utility for studying immune-mediated repair mechanisms.

The present study did not include histological assessment of muscle structure, including Z-line disruption, fiber necrosis, or inflammatory cell infiltration. Integrating histology will be essential to validate structural damage and fully characterize the EIMD profile induced by this model.

The selective oxidative stress activation without inflammatory responses positions this model as particularly valuable for antioxidant research. Many antioxidant interventions show conflicting results in EIMD studies, potentially because they interfere with beneficial inflammatory signaling required for adaptation. Our model could allow cleaner assessment of antioxidant effects without immune system confounding. Measuring additional oxidative stress markers will help strengthen the evidence that this model induces selective oxidative stress.

The delayed oxidative stress pattern may better model certain clinical conditions involving sustained low-grade muscle damage, such as age-related sarcopenia, compared to acute exercise injury models. This could provide clinically relevant insights into conditions characterized by oxidative stress without acute inflammation.

Future research should focus on protocol standardization through automated or mechanically controlled tail-pulling to reduce variability. The standardization of loading significantly affects the result. The 50-pull protocol was determined based on a pilot study. It can have a more intense exercise load with further methodologies. Earlier points (0–24 h) should also be examined to capture potential acute responses missed in our analysis. Although the triceps brachii is not the primary agonist during the grip task, secondary eccentric loading occurs as mice resist backward displacement during tail-pulling. The triceps was selected due to its larger size and high reproducibility in dissection. Nevertheless, future studies should target the digital and wrist flexor muscles, which are the actual agonists of the task, to more precisely evaluate muscle-specific inflammatory responses. Comparison studies directly contrasting this model with established approaches for specific research questions would help define optimal applications.

The unique characteristics suggest that this model may be most valuable for studying muscle regeneration mechanisms, antioxidant interventions, and oxidative stress roles in adaptation processes.

## 5. Conclusions

This study demonstrates that a small animal grip strength device can serve as a practical model for exercise-associated biochemical changes, particularly a reproducible elevation in plasma aldolase. While several oxidative stress-related parameters showed non-significant trends, these findings were insufficient to support oxidative stress induction. Therefore, this model primarily reflects mild muscle membrane disruption rather than classical inflammatory or oxidative responses. With its simplicity and reproducibility, this approach may complement existing EIMD models, although further work including controlled loading and histological confirmation is required.

## Figures and Tables

**Table 1:** Primer sequence for real-time PCR analysis.

Target Gene	Forward	Reverse
*Rn18s*	TTCTGGCCAACGGTCTAGACAAC	CCAGTGGTCTTGGTGTGCTGA
*Il6*	CTTGGGACTGATGCTGGTGACA	GCCTCCGACTTGTGAAGTGGTA
*Il1b*	TGCCACCTTTTGACAGTGATG	ATGTGCTGCTGCGAGATTTG
*Tnfa*	GTCCCCAAAGGGATGAGAAGT	TTTGCTACGACGTGGGCTAC
*Ly6g*	GGAGATAGAAGTTATTGTGGAC	TTGACAGCATTACCAGTGAT
*Il10*	ACATACTGCTAACCGACTCCT	GGCATCACTTCTACCAGGTAA
*Nfkb1*	GCCTCTAGTGAGAAGAACAA	GTGACCAACTGAACGATAAC
*K* *eap1*	ATGTTGACACGGAGGATTG	ACATTCTGCGGAGTTAGC
*Nfe2l2*	TTGCCACCGCCAGGACTACA	AACTTGTACCGCCTCGTCTGGA

*Rn18s*, 18s ribosomal RNA; *Tnfa*, tumor necrosis factor-α; *Il1b*, interleukin 1β; *Il6*, interleukin 6; *Nfkb1*, nuclear factor kappa B; *Nfe2l2*, nuclear factor (erythroid-derived 2)-like 2; *Keap1*, kelch-like ECH-associated protein 1; *Il10*, interleukin 10; *Ly6g*, lymphocyte antigen 6 family member G.

## Data Availability

The original contributions presented in this study are included in the article. Further inquiries can be directed to the corresponding author.

## References

[1] Proske U., Morgan D.L. (2001). Muscle damage from eccentric exercise: Mechanism, mechanical signs, adaptation and clinical applications. J. Physiol..

[2] Paulsen G., Mikkelsen U.R., Raastad T., Peake J.M. (2012). Leucocytes, cytokines and satellite cells: What role do they play in muscle damage and regeneration following eccentric exercise?. Exerc. Immunol. Rev..

[3] Mizumura K., Taguchi T. (2016). Delayed onset muscle soreness: Involvement of neurotrophic factors. J. Physiol. Sci..

[4] Nieman D.C., Dumke C.L., Henson D.A., McAnulty S.R., Gross S.J., Lind R.H. (2005). Muscle damage is linked to cytokine changes following a 160-km race. Brain Behav. Immun..

[5] Nosaka K., Newton M., Sacco P. (2002). Delayed-onset muscle soreness does not reflect the magnitude of eccentric exercise-induced muscle damage. Scand. J. Med. Sci. Sports.

[6] Isner-Horobeti M.E., Dufour S.P., Vautravers P., Geny B., Coudeyre E., Richard R. (2013). Eccentric exercise training: Modalities, applications and perspectives. Sports Med..

[7] Lavender A.P., Nosaka K. (2006). Changes in fluctuation of isometric force following eccentric and concentric exercise of the elbow flexors. Eur. J. Appl. Physiol..

[8] Proske U., Allen T.J. (2005). Damage to skeletal muscle from eccentric exercise. Exerc. Sport Sci. Rev..

[9] Fridén J., Lieber R.L. (1998). Segmental muscle fiber lesions after repetitive eccentric contractions. Cell Tissue Res..

[10] Suzuki K. (2019). Characterization of exercise-induced cytokine release, the impacts on the body, the mechanisms and modulations. Int. J. Sports Exerc. Med..

[11] Suzuki K., Tominaga T., Ruhee R.T., Ma S. (2020). Characterization and modulation of systemic inflammatory response to exhaustive exercise in relation to oxidative stress. Antioxidants.

[12] Ostrowski K., Rohde T., Asp S., Schjerling P., Pedersen B.K. (1999). Pro- and anti-inflammatory cytokine balance in strenuous exercise in humans. J. Physiol..

[13] Athanasiou N., Bogdanis G.C., Mastorakos G. (2023). Endocrine responses of the stress system to different types of exercise. Rev. Endocr. Metab. Disord..

[14] Bowtell J., Kelly V. (2019). Fruit-Derived Polyphenol Supplementation for Athlete Recovery and Performance. Sports Med..

[15] Tiidus P.M. (1998). Radical species in inflammation and overtraining. Can. J. Physiol. Pharmacol..

[16] Powers S.K., Deminice R., Ozdemir M., Yoshihara T., Bomkamp M.P., Hyatt H. (2020). Exercise-induced oxidative stress: Friend or foe?. J. Sport Health Sci..

[17] Pingitore A., Lima G.P., Mastorci F., Quinones A., Iervasi G., Vassalle C. (2015). Exercise and oxidative stress: Potential effects of antioxidant dietary strategies in sports. Nutrition.

[18] Armstrong R.B., Ogilvie R.W., Schwane J.A. (1983). Eccentric exercise-induced injury to rat skeletal muscle. J. Appl. Physiol. Respir. Environ. Exerc. Physiol..

[19] Kawashima M., Iguchi S., Fujita N., Miki A., Arakawa T. (2021). Structural Changes in Skeletal Muscle Fibers after Icing or Heating on Downhill Running in Mice. Kobe J. Med. Sci..

[20] Boykin J.R., Steiner J.L., Laskin G.R., Roberts M.D., Vied C., Willis C.R.G., Etheridge T., Gordon B.S. (2025). Comparative analysis of acute eccentric contraction-induced changes to the skeletal muscle transcriptome in young and aged mice and humans. Am. J. Physiol. Regul. Integr. Comp. Physiol..

[21] Hayashi K., Katanosaka K., Abe M., Yamanaka A., Nosaka K., Mizumura K., Taguchi T. (2017). Muscular mechanical hyperalgesia after lengthening contractions in rats depends on stretch velocity and range of motion. Eur. J. Pain.

[22] Pizza F.X., Peterson J.M., Baas J.H., Koh T.J. (2005). Neutrophils contribute to muscle injury and impair its resolution after lengthening contractions in mice. J. Physiol..

[23] Morton A.B., Norton C.E., Jacobsen N.L., Fernando C.A., Cornelison D.D.W., Segal S.S. (2019). Barium chloride injures myofibers through calcium-induced proteolysis with fragmentation of motor nerves and microvessels. Skelet. Muscle.

[24] Jansen E.H., Ruskovska T. (2013). Comparative analysis of serum (anti)oxidative status parameters in healthy persons. Int. J. Mol. Sci..

[25] Mandas A., Congiu M.G., Balestrieri C., Mereu A., Iorio E.L. (2008). Nutritional status and oxidative stress in an elderly Sardinian population. Med. J. Nutr. Metab..

[26] Trotti R., Carratelli M., Barbieri M., Micieli G., Bosone D., Rondanelli M., Bo P. (2001). Oxidative stress and a thrombophilic condition in alcoholics without severe liver disease. Haematologica.

[27] Casciola-Rosen L., Hall J.C., Mammen A.L., Christopher-Stine L., Rosen A. (2012). Isolated elevation of aldolase in the serum of myositis patients: A potential biomarker of damaged early regenerating muscle cells. Clin. Exp. Rheumatol..

[28] Baird M.F., Graham S.M., Baker J.S., Bickerstaff G.F. (2012). Creatine-kinase- and exercise-related muscle damage implications for muscle performance and recovery. J. Nutr. Metab..

[29] Clarkson P.M., Nosaka K., Braun B. (1992). Muscle function after exercise-induced muscle damage and rapid adaptation. Med. Sci. Sports Exerc..

[30] Sayers S.P., Clarkson P.M. (2003). Short-term immobilization after eccentric exercise. Part II: Creatine kinase and myoglobin. Med. Sci. Sports Exerc..

[31] Malaguti M., Angeloni C., Garatachea N., Baldini M., Leoncini E., Collado P.S., Teti G., Falconi M., Gonzalez-Gallego J., Hrelia S. (2009). Sulforaphane treatment protects skeletal muscle against damage induced by exhaustive exercise in rats. J. Appl. Physiol..

[32] Eppenberger H.M., Dawson D.M., Kaplan N.O. (1967). The comparative enzymology of creatine kinases. I. Isolation and characterization from chicken and rabbit tissues. J. Biol. Chem..

[33] Castellino F.J., Barker R. (1968). Examination of the dissociation of multichain proteins in guanidine hydrochloride by membrane osmometry. Biochemistry.

[34] Cannon J.G., Fielding R.A., Fiatarone M.A., Orencole S.F., Dinarello C.A., Evans W.J. (1989). Increased interleukin 1 beta in human skeletal muscle after exercise. Am. J. Physiol..

[35] Cannon J.G., St Pierre B.A. (1998). Cytokines in exertion-induced skeletal muscle injury. Mol. Cell. Biochem..

[36] Hirose L., Nosaka K., Newton M., Laveder A., Kano M., Peake J., Suzuki K. (2004). Changes in inflammatory mediators following eccentric exercise of the elbow flexors. Exerc. Immunol. Rev..

[37] Peake J.M., Nosaka K., Muthalib M., Suzuki K. (2006). Systemic inflammatory responses to maximal versus submaximal lengthening contractions of the elbow flexors. Exerc. Immunol. Rev..

[38] Brenner I.K., Natale V.M., Vasiliou P., Moldoveanu A.I., Shek P.N., Shephard R.J. (1999). Impact of three different types of exercise on components of the inflammatory response. Eur. J. Appl. Physiol. Occup. Physiol..

[39] Suzuki K., Totsuka M., Nakaji S., Yamada M., Kudoh S., Liu Q., Sugawara K., Yamaya K., Sato K. (1999). Endurance exercise causes interaction among stress hormones, cytokines, neutrophil dynamics, and muscle damage. J. Appl. Physiol..

[40] Kim M., Chun J., Jung H.A., Choi J.S., Kim Y.S. (2017). Capillarisin attenuates exercise-induced muscle damage through MAPK and NF-κB signaling. Phytomedicine.

[41] Ahmed S.M., Luo L., Namani A., Wang X.J., Tang X. (2017). Nrf2 signaling pathway: Pivotal roles in inflammation. Biochim. Biophys. Acta Mol. Basis Dis..

[42] Suzuki K., Nakaji S., Yamada M., Totsuka M., Sato K., Sugawara K. (2002). Systemic inflammatory response to exhaustive exercise. Cytokine kinetics. Exerc. Immunol. Rev..

